# Antioxidant and Anti-Osteoporotic Activities of Aromatic Compounds and Sterols from *Hericium erinaceum*

**DOI:** 10.3390/molecules22010108

**Published:** 2017-01-11

**Authors:** Wei Li, Sang Hyun Lee, Hae Dong Jang, Jin Yeul Ma, Young Ho Kim

**Affiliations:** 1 Korean Medicine (KM) Application Center, Korea Institute of Oriental Medicine, Daegu 41062, Korea; liwei1986@kiom.re.kr; 2 Department of Food and Nutrition, Hannam University, Daejeon 34430, Korea; shlee@hnu.ac.kr; 3 College of Pharmacy, Chungnam National University, Daejeon 34134, Korea

**Keywords:** *Hericium erinaceum*, hericiaceae, antioxidant, anti-osteoporosis, sterol, aromatic compound

## Abstract

*Hericium erinaceum*, commonly called lion’s mane mushroom, is a traditional edible mushroom widely used in culinary applications and herbal medicines in East Asian countries. In this study, a new sterol, cerevisterol 6-cinnamate (**6**), was isolated from the fruiting bodies of *H. erinaceum* together with five aromatic compounds **1**–**5** and five sterols **7**–**11**. The chemical structures of these compounds were elucidated using chemical and physical methods and comparison of HRESIMS, ^1^D-NMR (^1^H, ^13^C, and DEPT) and 2D-NMR (COSY, HMQC, HMBC, and NOESY) spectra with previously reported data. The antioxidant and anti-osteoporotic activities of extracts and the isolated compounds **1**–**11** were investigated. All compounds exhibited peroxyl radical-scavenging capacity but only compounds **1**, **3**, and **4** showed potent reducing capacity. Moreover, compounds **1**, **2**, **4**, and **5** showed moderate effects on cellular antioxidant activity and inhibited the receptor activator of nuclear factor κB ligand (RANKL)-induced osteoclastic differentiation. These results suggested that *H. erinaceum* could be utilized in the development of natural antioxidant and anti-osteoporotic nutraceuticals and functional foods.

## 1. Introduction

Oxidative stress is caused by an imbalance in the generation of reactive oxygen species (ROS) and the activity of antioxidant defenses. Severe oxidative stress has been implicated in many chronic and degenerative diseases, including osteoporosis, cancer, aging, and neurodegenerative diseases such as Alzheimer’s disease, Parkinson’s disease, and amyotrophic lateral sclerosis [1]. Several natural antioxidants have been reported, including flavonoids, coumarins, anthocyanins, and phenolic acids. Some natural flavonoids and coumarins with antioxidant activity, including scopoletin and baicalein, have been found to exert potent anti-osteoporotic effects through the suppression of osteoclast formation and tartrate-resistant acid phosphatase (TRAP) [2,3,4]. Therefore, interest in novel natural antioxidants has increased. Osteoclasts are tissue-specific macrophage polykaryons formed by the differentiation of monocyte/macrophage precursor cells at or near the bone surface in response to osteoclastogenic factors, particularly the receptor activator of nuclear factor κB ligand (RANKL), which performs important functions in osteoclast differentiation, including mediation and activation [5,6]. This developmental process results in TRAP expression in the differentiating cells, followed by their fusion to produce multinucleated cells capable of resorbing a mineralized matrix [7].

In our continuing search for antioxidant and anti-osteoporotic components from natural sources, we found that the MeOH extract of *Hericium erinaceum* showed potent effects. *H. erinaceum*, commonly called lion’s mane mushroom, is an edible medicinal fungus found in China, Korea, and Japan, and is used as a raw material in a wide range of East Asian foods. Its fruiting bodies are used to treat gastric ulcers, chronic gastritis, and other digestive tract-related diseases [8]. The constituents of *H. erinaceum* include polysaccharides, heteropolysaccharides, diterpenoids, and aromatic compounds [9]. Previous pharmacological studies on compounds isolated from *H. erinaceum* also showed their stimulatory effects on nerve growth factor synthesis and antitumor activity [10,11]. However, no studies have investigated the active components of *H. erinaceum* with antioxidant and anti-osteoporotic activities. In this study, bioassay-guided isolation of the CHCl_3_ fraction of the MeOH extract of *H. erinaceum* yielded 11 compounds **1**–**11**, which were evaluated for antioxidant activity using oxygen radical absorbance capacity (ORAC), cupric ion reducing antioxidant capacity (CUPRAC), and cellular antioxidant capacity (CAC) assays. Moreover, the anti-osteoporotic activities of these compounds were evaluated by investigating their inhibitory effects on osteoclastic differentiation.

## 2. Results and Discussion

**Figure 1:**
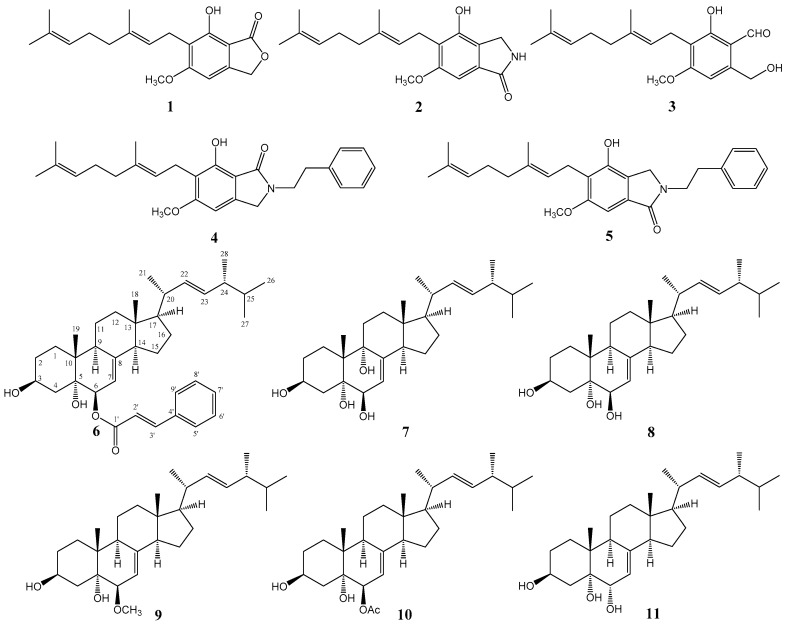
Structures of compounds **1**–**11** isolated from cultures of *H. erinaceum*.

Using combined chromatographic separations, 11 compounds, including five aromatic compounds **1**–**5** and six sterols **6**–**11**, one of which is new (cerevisterol 6-cinnamate, compound **6**), were isolated from the CHCl_3_ fraction of the MeOH extract of *H. erinaceum* fruiting bodies (Figure 1). The known compounds were identified as hericenone J (**1**) [12], *N*-dephenylethyl isohericerin (**2**), 4-[3′,7′-dimethyl-2′,6′-octadienyl]-2-formyl-3-hydroxy-5-methyoxy-benzylalcohol (**3**) [13], isohericerin (**4**) [14], hericerin (**5**) [14], (22*E*,24*R*)-ergosta-7,22-diene-3β,5α,6β,9α-tetraol (**7**) [15], cerevisterol (**8**) [16], blazein (**9**) [16], 3β,5α-dihydroxy-6β-acetoxy-ergosta-7,22-diene (**10**) [17], and ergosta-7,22-diene-3β,5α,6α-triol (**11**) [18]. The purity of all isolated compounds was confirmed to be >95% using HPLC. Compound structures were elucidated by comparison of spectroscopic data with reported values. Six compounds (**6**–**11**) were isolated from *H. erinaceum* for the first time.

**Figure 2:**
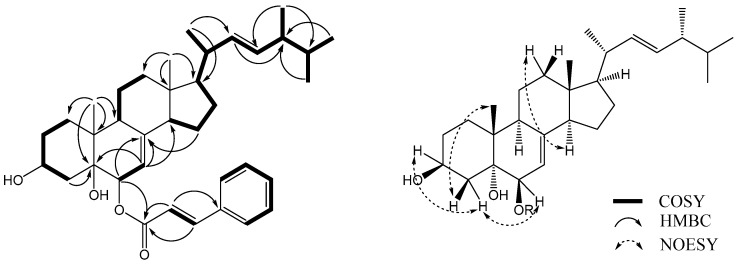
Key ^1^H-^1^H COSY, HMBC, and NOESY correlations of compound **6**.

Compound **6** was obtained as a yellowish oil. The molecular formula C_37_H_52_O_4_ was assigned based on a pseudomolecular ion peak at *m*/*z* 561.3944 [M + H]^+^ (calcd. 561.3938) in the HR-ESI-MS spectrum. The ^1^H-NMR spectrum of **6** (Table 1) contained peaks corresponding to six methyl groups [δ_H_ 0.53 (s, Me-18), 0.76 (d, *J* = 6.5 Hz, Me-26, 27), 0.84 (d, *J* = 6.7 Hz, Me-28), 0.95 (d, *J* = 6.5 Hz, Me-21), and 1.08 (s, Me-19)], two oxymethines [δ_H_ 4.02 (m, H-3α) and 4.93 (d, *J* = 5.4 Hz, H-6α)], and three olefinic protons at δ_H_ 5.09 (dd, *J* = 15.3, 7.7 Hz, H-22), 5.14 (dd, *J* = 15.3, 7.0 Hz, H-23), and 5.26 (d, *J* = 5.4 Hz, H-7). Additionally, cinnamic acid residue signals were observed at δ_H_ 6.33 (d, *J* = 16.0 Hz, H-2′), 7.30-7.31 (m, H-6′, 7′, 8′), 7.46 (d, *J* = 8.0 Hz, H-5′, 9′), and 7.58 (d, *J* = 16.0 Hz, H-3′). The ^13^C-NMR spectrum (Table 1) contained 28 resonances corresponding to the sterol moiety, which were assigned to six methyl groups (δ_C_ 12.5, 17.6, 18.5, 19.7, 20.3, and 21.2), seven methylene groups (δ_C_ 22.2, 22.8, 27.9, 30.7, 32.6, 39.3, and 39.4), eight sp^3^ methine groups (δ_C_ 33.1, 40.4, 43.4, 55.0, 56.0, 67.5, and 73.5), three sp^2^ methine groups (δ_C_ 114.3, 132.2, and 135.4), and four quaternary carbon atoms (δ_C_ 37.3, 43.9, 75.5, and 145.9). Signals from the cinnamic acid residue revealed a carbonyl group [δ_C_ 166.4 (C-1′)], two olefinic carbon atoms [δ_C_ 118.4 (C-2′) and 145.1 (C-3′)] and six aromatic carbon atoms [δ_C_ 128.2 (C-5′, 9′), 128.9 (C-6′, 8′), 130.4 (C-7′), and 134.4 (C-4′)]. The above data showed that the sterol moiety **6** was a Δ^7^-ergostane derivative, which was similar to compound **8** (cerevisterol) [19], but had a cinnamic acid residue attached at C-6. This finding was supported by a key HMBC correlation between H-6α (δ_H_ 4.93) and C-1′ (δ_C_ 166.4) (Figure 2). Alkaline hydrolysis of **6** with 0.5% NaOH yielded cerevisterol and cinnamic acid, which were identified by TLC analysis (CHCl_3_–acetone: 2:1; *R*_f_ = 0.35, 0.80) with known standards (see Supplementary Materials).

The relative configuration of **6** was determined using a NOESY NMR experiment and empirical pyridine-induced deshielding effects. NOE correlations were observed between H-6α (δ_H_ 4.93) and H_eq_-4 (δ_H_ 1.69), H-3*α* (δ_H_ 4.02) and H_eq_-4 (δ_H_ 1.69), and Me-19 (δ_H_ 1.08) and H_ax_-4 (δ_H_ 1.87), which indicated that the two hydroxyl groups were in 3β,6β-orientations (Figure 2) [18,19]. The chemical shifts of H_ax_-3, H_ax_-4, H-6, and Me-19 measured in pyridine-*d*_5_ were shifted downfield, which indicated the presence of a 3β,5α,6β-trihydroxylated system in compound **6** [15,20]. A comparison of the chemical shifts of C-26, C-27, and C-28 of **6** with those of compound **8** established a 24*R* configuration in **6** [21]. The large coupling constant (15.3 Hz) between H-22 and H-23 was indicative of an *E* configuration for the double bond [22]. Therefore, compound **6** was identified as cerevisterol 6-cinnamate [(22*E*,24*R*)-ergosta-7,22-dien-3β,5α,6β-triol 6-cinnamate].

**Figure 3:**
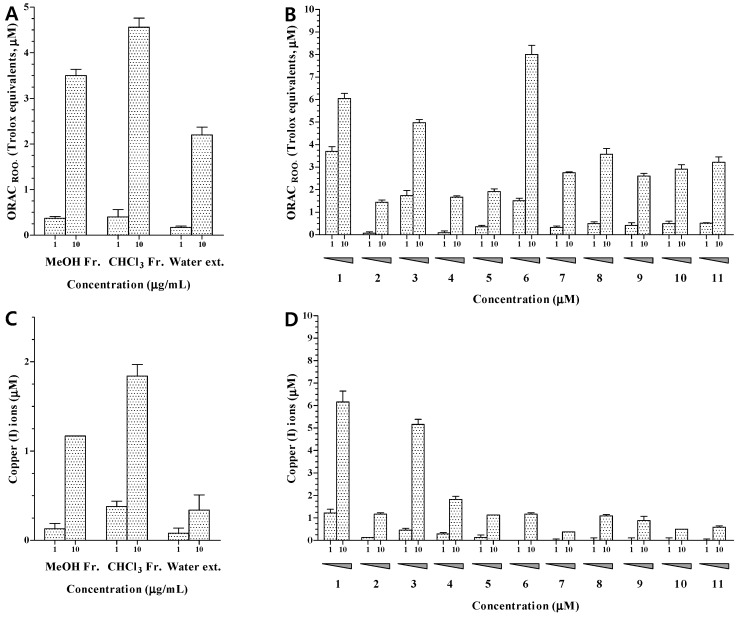
Peroxyl radical-scavenging capacity ((**A**,**B**)) and reducing capacity ((**C**,**D**)) of the extracts and compounds **1**–**11** from *H. erinaceum*. Data are expressed as the mean ± standard deviation of three individual experiments. Statistical significance is determined by one-way ANOVA followed by Dunnett’s multiple comparison test (*p* < 0.05).

The MeOH extract and CHCl_3_ and water fractions of *H. erinaceum* were evaluated for their antioxidant activity at concentrations of 1.0 and 10.0 μg/mL. The MeOH extract and CHCl_3_ fraction showed potent effects at 10.0 μg/mL in the ORAC and CUPRAC assays (Figure 3A,C). In order to identify the active antioxidant and anti-osteoporotic components of *H. erinaceum*, 11 compounds (**1**–**11**) from the CHCl_3_ fraction were evaluated using ORAC, CUPRAC, CAC, and TRAP assays. The antioxidant activities of compounds **1**–**11** were investigated by ORAC and CUPRAC assays. The ORAC assay is based on hydrogen atom transfer while the CUPRAC reducing capacity assay depends on single electron transfer [23]. The results showed that compounds **1**, **3**, and **6** had potent peroxyl radical-scavenging activities at 10 μM with ORAC values of 6.05, 4.98, and 8.01, respectively. Other compounds showed negligible activities with ORAC values below 4.00 (Figure 3B). In structure-activity analysis of the isolated compounds, the aromatic compounds **1** and **3** showed more potent activity than the other aromatic compounds (**2**, **4**, and **5**). The same hydroxyl group is present in the aromatic ring but compounds **2**, **4**, and **5** also contain a nitrogen atom. Therefore, it appears that the nitrogen atom in the aromatic ring exerts a negative influence on peroxyl radical-scavenging activity. Compound **6** exhibited the strongest activity among the isolated sterols (**6**–**11**). Based on structural analysis, the cinnamic acid residue at C-6 seems to be an important element for peroxyl radical-scavenging activity.

The ability to transfer single electrons was examined by reducing capacity assay. The reducing capacity of all compounds was evaluated based on the production of Cu(I) ions from Cu(II) ions in the presence of each isolated compound. The results revealed that only compounds **1** and **3** exhibited an obvious reducing capacity at 10.0 μM, with CUPRAC values of 6.16 and 5.16, respectively (Figure 3D). These results indicated that the ability of compounds **1** and **3** to donate hydrogen atoms or electrons to peroxyl radicals, and to convert them into relatively stable compounds, may contribute to their peroxyl radical-scavenging capacity.

**Figure 4:**
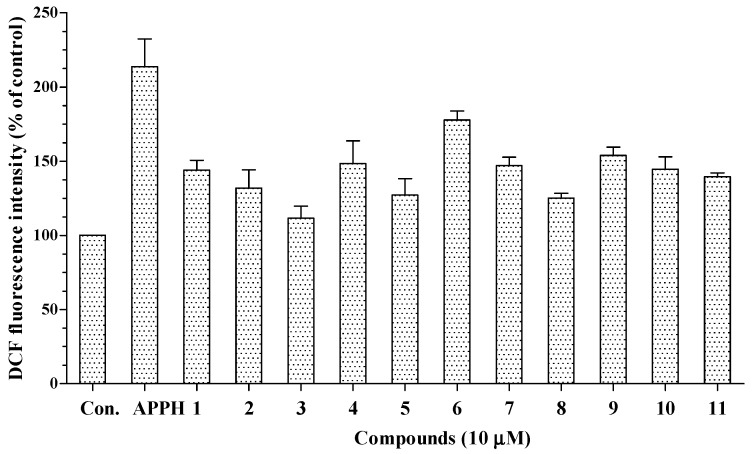
Cellular antioxidant capacity of compounds **1**–**11** against oxidative stress induced by 2,2'-azobisisobutyramidinium chloride (APPH) (Con.: control). Data are expressed as percentages of the value of untreated cells (mean ± standard deviation of three individual experiments). Statistical significance is determined by one-way ANOVA followed by Dunnett’s multiple comparison test (*p* < 0.05).

The intracellular antioxidant capacities of compounds **1**–**11** were investigated by a CAC assay. First, cell viability was tested by an MTT assay. The results showed that compounds **1**–**11** were not significantly cytotoxic to HepG2 cells at the tested concentrations. Following treatment with AAPH, intracellular oxidative stress in HepG2 cells increased to 213.8% of the control value. As shown in Figure 4, all 11 compounds decreased the intracellular oxidative stress caused by AAPH to 111.6%–177.7% of the control value. Thus, all the isolated compounds may permeate the cell membrane and suppress the intracellular oxidative stress induced by peroxyl radicals.

**Figure 5:**
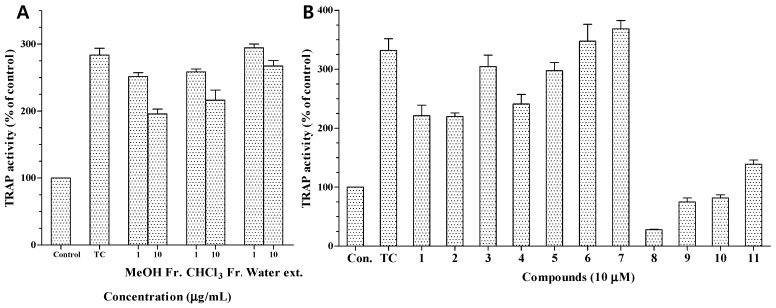
Inhibitory effects of the extracts (**A**) and compounds **1**–**11** (**B**) on TRAP activity of osteoclastic RAW 264.7 cells. Data are expressed as percentages of the values obtained with untreated cells. (Con.: control, which was not treated; TC: treated control, which was treated with RANKL).

The MeOH extract and CHCl_3_ fraction also exhibited moderate TRAP activity at 10.0 μg/mL (Figure 5A). We also investigated the anti-osteoporotic activity of compounds **1**–**11** by evaluating their inhibitory effects on TRAP activity in osteoclasts differentiated from RAW 264.7 cells. Osteoclast differentiation was induced by RANKL, a key cytokine regulating osteoclastogenesis and bone resorption. RANKL treatment strongly induced osteoclast formation from RAW 264.7 pre-osteoclasts and markedly increased TRAP activity [24]. TRAP is highly expressed in osteoclasts and is widely used as a phenotypic marker of osteoclasts. Based on the reduction in TRAP activity, the inhibitory activities of compounds **1**–**11** against osteoporosis are shown in Figure 5. At a concentration of 10 μM, compounds **8**–**11** showed strong inhibition of RANKL-induced TRAP activity, reducing it to 28.1%, 74.8%, 81.8%, and 138.9%, respectively, from a value of 332% in RANKL-treated control cells. The potent inhibitory effects of compounds **8**–**11** may be due to their strong cytotoxicity. In contrast, compounds **1**, **2**, **4**, and **5** showed moderate inhibition at 10 μM (Figure 5B), which indicated that the presence of an α,β-unsaturated carbonyl moiety in the structure may be involved in the inhibitory effects on the TRAP activity of osteoclasts.

Recent studies have suggested that intracellular antioxidant activity related to ROS scavenging may be responsible for increased osteoblast differentiation in MC3T3-E1 pre-osteoblastic cells and suppression of the differentiation of RAW 264.7 pre-osteoclast cells into osteoclasts [2,25]. However, a comparison of the anti-osteoporotic and antioxidant activities of compounds **1**–**11** indicated their anti-osteoporotic activity appeared to be associated with cellular antioxidant activity instead of in vitro antioxidant activity, but was not proportional to cellular antioxidant activity. These results can be explained by the different plausible mechanisms that may contribute to cellular antioxidant activities, such as the induction of phase II detoxifying and antioxidant enzymes as an indirect source of antioxidant activity. In addition, the important role of the α,β-unsaturated carbonyl moiety in the indirect cellular antioxidant activity of these compounds (**1**, **2**, **4**, and **5**) can be ignored. However, more research is required to determine whether the potent anti-osteoporotic activities of compounds **1**, **2**, **4**, and **5** result from indirect antioxidant activity.

## 3. Materials and Methods

### 3.1. General Information

Optical rotations were determined using a DIP-370 automatic polarimeter (Jasco, Easton, MD, USA). The FT-IR spectra were measured using a Jasco Report-100 infrared spectrometer; The NMR spectra were recorded using an ECA 600 spectrometer (^1^H, 600 MHz; ^13^C, 150 MHz, JEOL, Tokyo, Japan). Mass spectra were recorded on an LCQ advantage trap mass spectrometer (Thermo Finnigan, San Jose, CA, USA) equipped with an electrospray ionization (ESI) source. High-resolution electrospray ionization mass spectra (HR-ESI-MS) were obtained using a 6530 Accurate-Mass Q-TOF LC/MS system (Agilent, Santa Clara, CA, USA). Column chromatography was performed using silica gel (Kieselgel 60, 70–230, and 230–400 mesh, Merck, Darmstadt, Germany) and YMC RP-18 resins (Merck), and thin layer chromatography (TLC) was performed using pre-coated silica-gel 60 F_254_ and RP-18 F_254_S plates (both 0.25 mm, Merck).

### 3.2. Fungal Material

Dried fruiting bodies of *H. erinaceum* were purchased from an herbal market in Kumsan, Chungnam Province, Korea in August 2013. The identity of the specimens was confirmed by one of authors (Young Ho Kim). A voucher specimen (CNU 13110) was deposited at the Herbarium of the College of Pharmacy at Chungnam National University.

### 3.3. Fermentation, Extraction and Isolation

Dried fruiting bodies (2.5 kg) of *H. erinaceum* were extracted with MeOH (5 L, × 3) under reflux. The MeOH extract (320.0 g) was suspended in water (5.0 L) and partitioned with CHCl_3_ (5.0 L) yielding CHCl_3_ (90.0 g) and water (220.0 g) fractions. The CHCl_3_ fraction (90.0 g) was subjected to silica gel (5.0 cm × 30 cm) column chromatography with a gradient of *n*-hexane–EtOAc–MeOH (25:1:0, 9:1:0, 5:1:0, 2.5:1:0, 1:1:0.1, 1:1:0.3, 0.5:1:0.5; 4 L for each step) to give eight fractions (Fr. 1A–1H).

Fraction 1C was separated on a silica gel column with a gradient of *n*-hexane–EtOAc (20:1 to 10:1, 10 L) to give 11 sub-fractions (Fr. 1C-1–1C-11). Fraction 1C-8 was subjected to YMC (1.0 × 80 cm) column chromatography eluted with a MeOH–acetone–H_2_O (3:3:1, 4:4:1, 6:6:1; 1.2 L for each step) gradient to give compounds **1** (43.6 mg) and **5** (86.2 mg). Fraction 1D was subjected to silica gel (2.5 cm × 30 cm) column chromatography with a gradient of *n*-hexane–EtOAc–MeOH (8:1:0.15, 6:1:0.15, 4:1:0.15, 3:1:0.15, 2:1:0.15, 1.5:1:0.15; 2.5 L for each step) to give 10 fractions (Fr. 1D-1–1D-10). Fraction 1D-7 was subjected to YMC (1.5 cm × 80 cm) column chromatography eluting with a MeOH–acetone–H_2_O (1.5:1:1, 3:1.5:1, 6:3.5:1, 9:5:1; 1.0 L for each step) gradient to give compound **3** (21.4 mg). Fraction 1D-8 was subjected to silica gel (1.0 cm × 80 cm) column chromatography with a *n*-hexane–EtOAc–acetone (3:1:0.15; 750 mL) mixture as eluent to give compound **4** (13.4 mg). Fraction 1D-9 was subjected to YMC (2.5 cm × 80 cm) column chromatography eluting with MeOH–acetone–H_2_O (1:1:1, 2:2:1, 3:3:1, 5:5:1, 8:8:1, 10:10:1; 3 L for each step) to give eight sub-fractions (Fr. 1D-9-A–1D-9-H). Fraction 1D-9-E was subjected to silica gel (1.5 cm × 80 cm) column chromatography with a CHCl_3_–acetone (7:1, 9:1, 12:1; 1 L for each step) eluent to give compounds **9** (12.0 mg), **10** (7.0 mg), and **11** (22.0 mg). 1D-10 was subjected to YMC (2.5 cm × 80 cm) column chromatography eluting with MeOH–H_2_O (1:1, 2:1, 3:1, 5:1, 7:1, 9:1, 11:1, 13:1, 15:1; 2.5 L for each step) to give seven sub-fractions (Fr. 1D-10-A–1D-10-G) and compound **7** (42.0 mg). Fraction 1D-10-F was subjected to silica gel (1.0 cm × 80 cm) column chromatography with CHCl_3_–acetone (2.3:1; 1 L) as elution solvent to give compounds **6** (7.4 mg) and **8** (19.0 mg). Fraction 1E was separated using YMC (1.0 cm × 80 cm) column chromatography with MeOH–H_2_O mixtures (1:1, 3:1, 5:1, 7.5:1, 9:1, 15:1; 300 mL for each step) as eluent to give compound **2** (11.8 mg).

### 3.4. Product Characterization

*Cerevisterol 6-cinnamate* (**6**): Yellowish oil; C_12_H_12_O_5_; [α]${}_{D}^{25}$: −21.8 (*c* 0.2, MeOH); ^1^H-NMR (CDCl_3_, 600 MHz) and ^13^C-NMR data (CDCl_3_, 150 MHz), see Table 1; HR-ESI-MS (C_37_H_52_O_4_): *m*/*z* 561.3944 [M + H]^+^ (calcd. 561.3938).

### 3.5. Cell Toxicity Assay

Cell-Counting Kit (CCK)-8 (Dojindo, Kumamoto, Japan) was used to analyze the effect of compounds on cell toxicity according to the manufacturer’s instructions. Cells were cultured overnight in a 96-well plate (~1 × 10^4^ cells/well). Cell toxicity was assessed after the addition of compounds on dose-dependent manner. After 24 h of treatment, 10 μL of the CCK-8 solution was added to triplicate wells, and incubated for 1 h. Absorbance was measured at 450 nm to determine viable cell numbers in wells.

### 3.6. Oxygen Radical Absorbance Capacity (ORAC) Assay

The assay was performed on a Tecan GENios multi-functional plate reader (Tecan, Salzburg, Austria) with fluorescent filters using excitation and emission wavelengths of 485 and 535 nm, respectively. In the final assay mixture, 40 nM fluorescein was used as a target of free radical attack with 20 mM 2,2-azobis dihydrochloride (AAPH) as a peroxyl radical generator [26]. Trolox (1.0 μM) was used as a control standard and prepared fresh daily. The analyzer was programmed to record the fluorescence of fluorescein every 2 min after the addition of AAPH. Fluorescein (40 nM) in potassium phosphate buffer was used as the blank. All fluorescence measurements were expressed relative to the initial reading. Final results were calculated based on the difference in area under the fluorescence decay curve between the blank and each sample. ORAC_ROO·_ was expressed as micromoles of Trolox equivalents (TE). One ORAC unit is equivalent to the net protection area of 1.0 μM of Trolox.

### 3.7. Reduction Capacity

The reducing abilities of isolated compounds were determined according to the CUPRAC assay [27]. A total of 40 μL of different concentrations of the compounds in distilled water were mixed with 160 μL of a mixture containing 0.5 mM CuCl_2_ and 0.75 mM neocuproine in 10 mM phosphate buffer, pH 7.4. The absorbance was measured using a micro-plate reader at 454 nm for 1 h. The increase in the absorbance of the reaction mixture was indicative of increased reducing power. Distilled water was used as the blank.

### 3.8. Cellular Antioxidant Capacity

Cellular oxidative stress due to ROS generated by AAPH was measured spectrofluorometrically using the DCFH-DA method [28]. DCFH-DA diffuses through the cell membrane and is enzymatically hydrolyzed by intracellular esterase to non-fluorescent DCFH, which is rapidly oxidized to highly fluorescent DCF in the presence of ROS. HepG2 cells were first cultured in 96-well plates (5 × 10^5^/mL) with DMEM for 24 h. After the cells were incubated with different concentrations of extracts and isolated compounds dissolved in DMSO for 30 min, the medium was discarded, and the wells were gently washed twice with PBS. HBSS, which is fluorescently stable, was then added to each well instead of normal medium, and AAPH was used as an oxidative stress inducer. After the cells were treated with 60 μM AAPH for 30 min, DCFHDA was added to the culture plates at a final concentration of 40 μM and incubated for 30 min at 37 °C in the dark. Trolox (10 μM) was used as the positive control. After the incubation, the cells were washed with HBSS, and DCF fluorescence intensity was measured at an excitation wavelength of 485 nm and an emission wavelength of 535 nm using a Tecan GENios fluorometric plate reader. DMEM medium was used as the control.

### 3.9. Tartrate-resistant Acid Phosphatase (TRAP) Activity

RAW264.7 cells were seeded in 96-well plates (1 × 10^4^ cells/well) containing DMEM medium plus 10% fetal bovine serum (FBS) and the medium was replaced with test samples in a differentiation medium containing 50 ng/mL RANKL. After differentiating the RAW 264.7 cells into osteoclasts for 5 days, the medium was removed, and the cell monolayer was gently washed twice using ice-cold PBS. The cells were fixed in 3.5% formaldehyde for 10 min and ethanol–acetone (1:1) for 1 min. Subsequently, the dried cells were incubated in 50 mM citrate buffer (pH 4.5) containing 10 mM sodium tartrate and 6 mM p-nitrophenylphosphate (PNPP). After 1 h incubation, the reaction mixtures were transferred to new well plates containing an equal volume of 0.1 N NaOH. Absorbance was measured at 408 nm using an ELISA reader. Control was treated with only DMEM medium and treated control was treated with DMEM medium containing 50 ng/mL RANKL. TRAP activity was expressed as a percentage of the control.

## 4. Conclusions

In this study, a new sterol (cerevisterol 6-cinnamate; **6**), together with 10 known compounds **1**–**5** and **7**–**11** were isolated from the fruiting bodies of *H. erinaceum*. All compounds exhibited peroxyl radical-scavenging capacity, while compounds **1**, **3**, and **4** showed reducing capacity too. Moreover, some sterols showed moderate effects on cellular antioxidant activity and inhibited RANKL-induced osteoclastic differentiation. To the best of our knowledge, this is the first report on the antioxidant and anti-osteoporotic activities of aromatic compounds and sterol components from *H. erinaceum*. These results provide a scientific basis for the development of novel antioxidant and anti-osteoporotic agents from this mushroom.

## Figures and Tables

**Table 1:** ^1^H (600 MHz) and ^13^C-NMR (150 MHz) spectroscopic data of compound **6** (CDCl_3_, δ (ppm), *J* (Hz)).

Pos.	δ_H_	δ_C_	Pos.	δ_H_	δ_C_
**1**	1.57, m	32.6	**20**	1.99, m	40.4
	1.97, m				
**2**	1.41, m	30.7	**21**	0.95, d, 6.5	21.2
**3**	4.02, m	67.5	**22**	5.09, dd, 15.3, 7.7	135.4
**4**	1.69, m	39.4	**23**	5.14, dd, 15.3, 7.0	132.2
	1.87, m				
**5**	-	75.5	**24**	1.80, m	42.9
**6**	4.93, d, 5.4	73.5	**25**	1.40, m	33.1
**7**	5.26, d, 5.4	114.3	**26**	0.76, d, 6.5	20.3
**8**	-	145.9	**27**	0.76, d, 6.5	19.7
**9**	1.94, m	43.4	**28**	0.84, d, 6.7	17.6
**10**	-	37.3	**1′**	-	166.4
**11**	1.55, m	22.2	**2′**	6.33, d, 16.0	118.4
**12**	1.27, m	39.3	**3′**	7.58, d, 16.0	145.1
	1.68, m				
**13**	-	43.9	**4′**	-	134.4
**14**	1.87, m	55.0	**5′**	7.46, d, 8.0	128.2
**15**	1.35, m	22.8	**6′**	7.30–7.31, m	128.9
	1.51, m				
**16**	1.21, m	27.9	**7′**	7.30–7.31, m	130.4
	1.67, m				
**17**	1.22, m	56.0	**8′**	7.30–7.31, m	128.9
**18**	0.53, s	12.5	**9′**	7.46, d, 8.0	128.2
**19**	1.08, s	18.5			

## Supplementary Materials

The following are available online at: http://www.mdpi.com/1420-3049/22/1/108/s1, The NMR (^1^D and ^2^D) data of compound **6** are available as supporting information.
