# Supplementary material for: Antioxidant and Anti-Osteoporotic Activities of Aromatic Compounds and Sterols from Hericium erinaceum
Source: Molecules. 2017 Jan 11;22(1):108. doi: 10.3390/molecules22010108 (PMC6155785; doi:10.3390/molecules22010108)
Supplement: Supplementary file 1 [file molecules-22-00108-s001.pdf]

Supplementary Materials: Antioxidant and Anti-Osteoporotic Activities of Aromatic Compounds and Sterols from *Hericium erinaceum*

Wei Li, Sang Hyun Lee, Hae Dong Jang, Jin Yeul Ma and Young Ho Kim

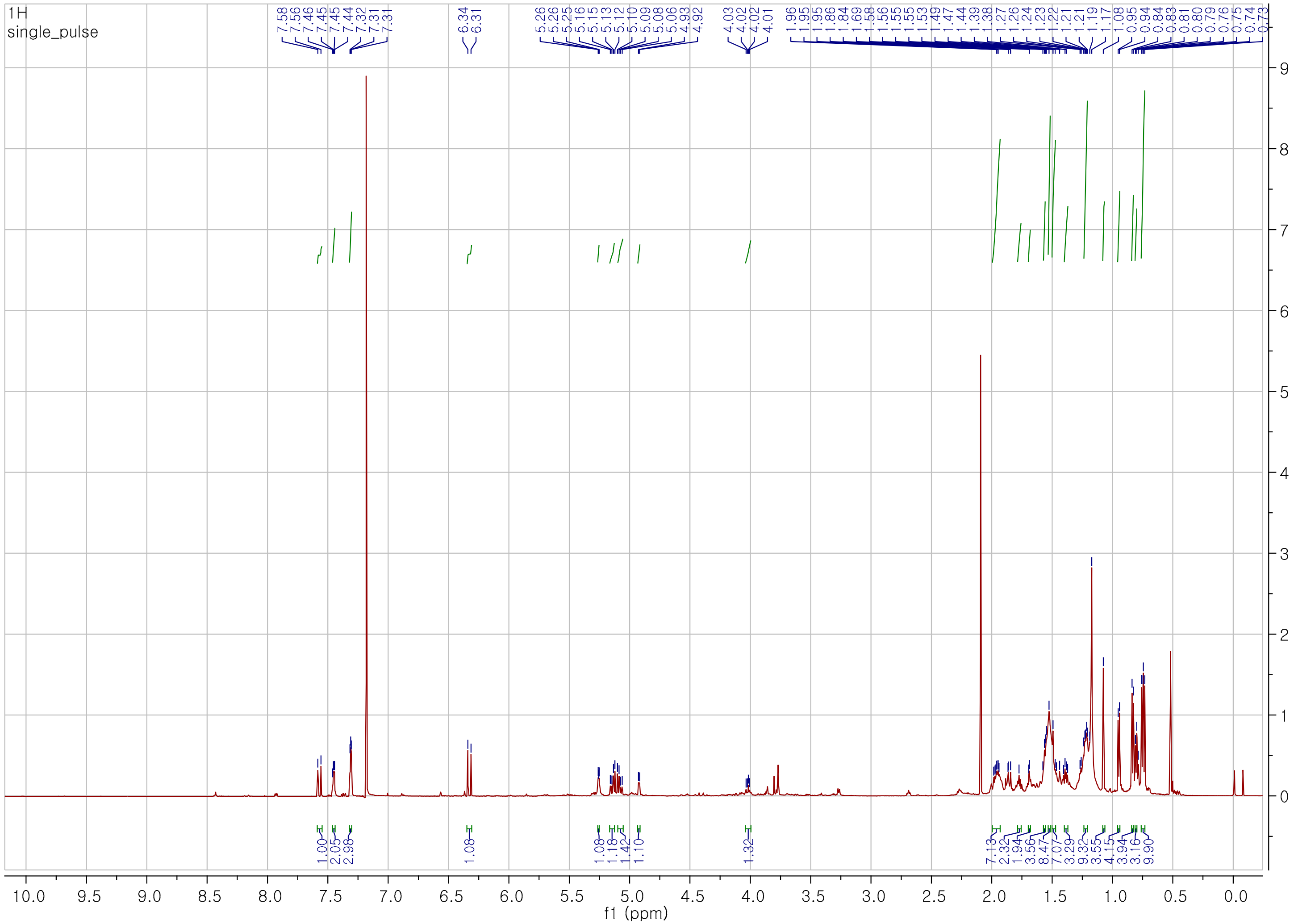

Figure S1. ^{1}H-NMR spectrum of compound **6** (600 MHz, $CDCl_3$).

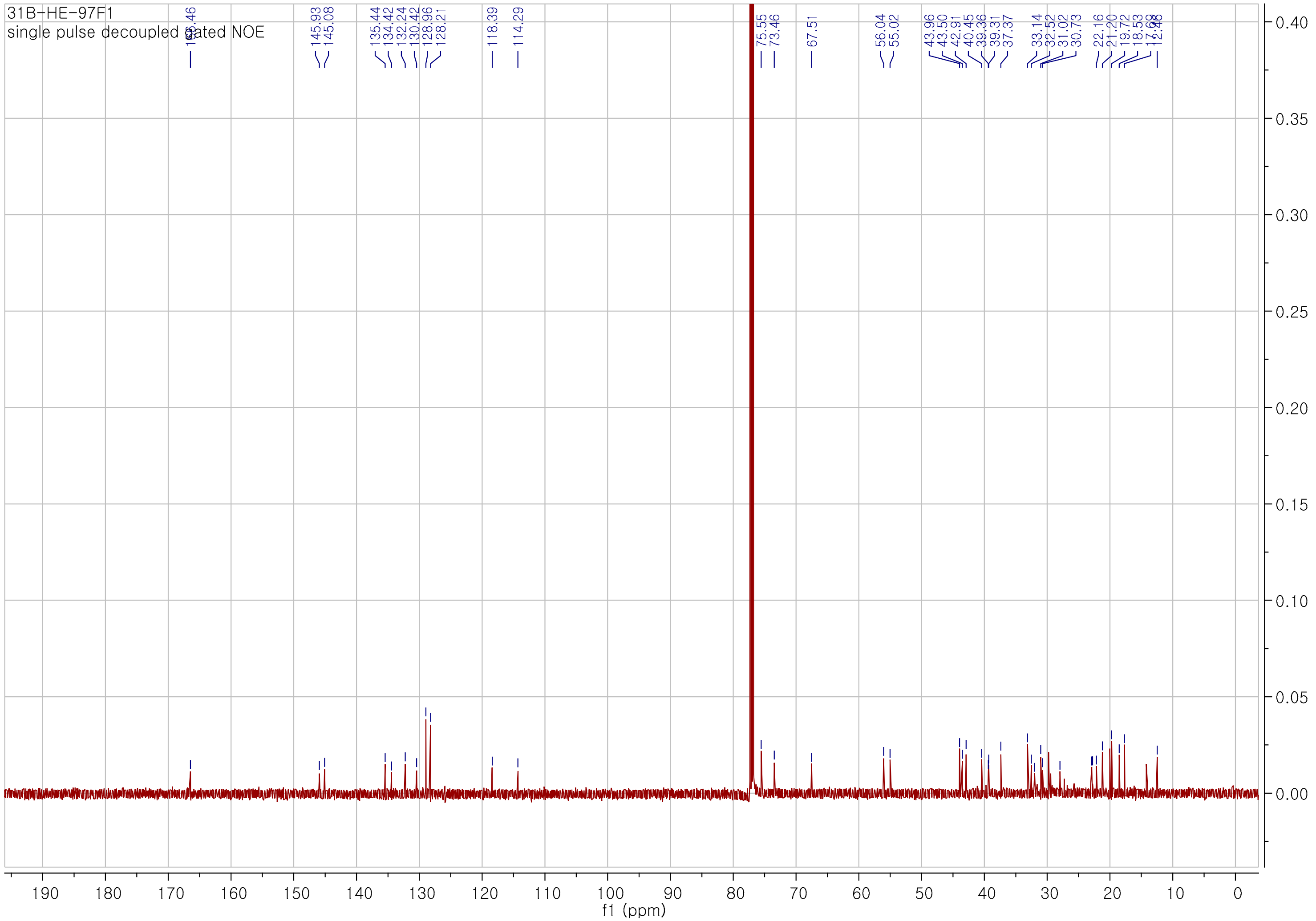

Figure S2. ^{13}C-NMR spectrum of compound **6** (150 MHz, $CDCl_3$).

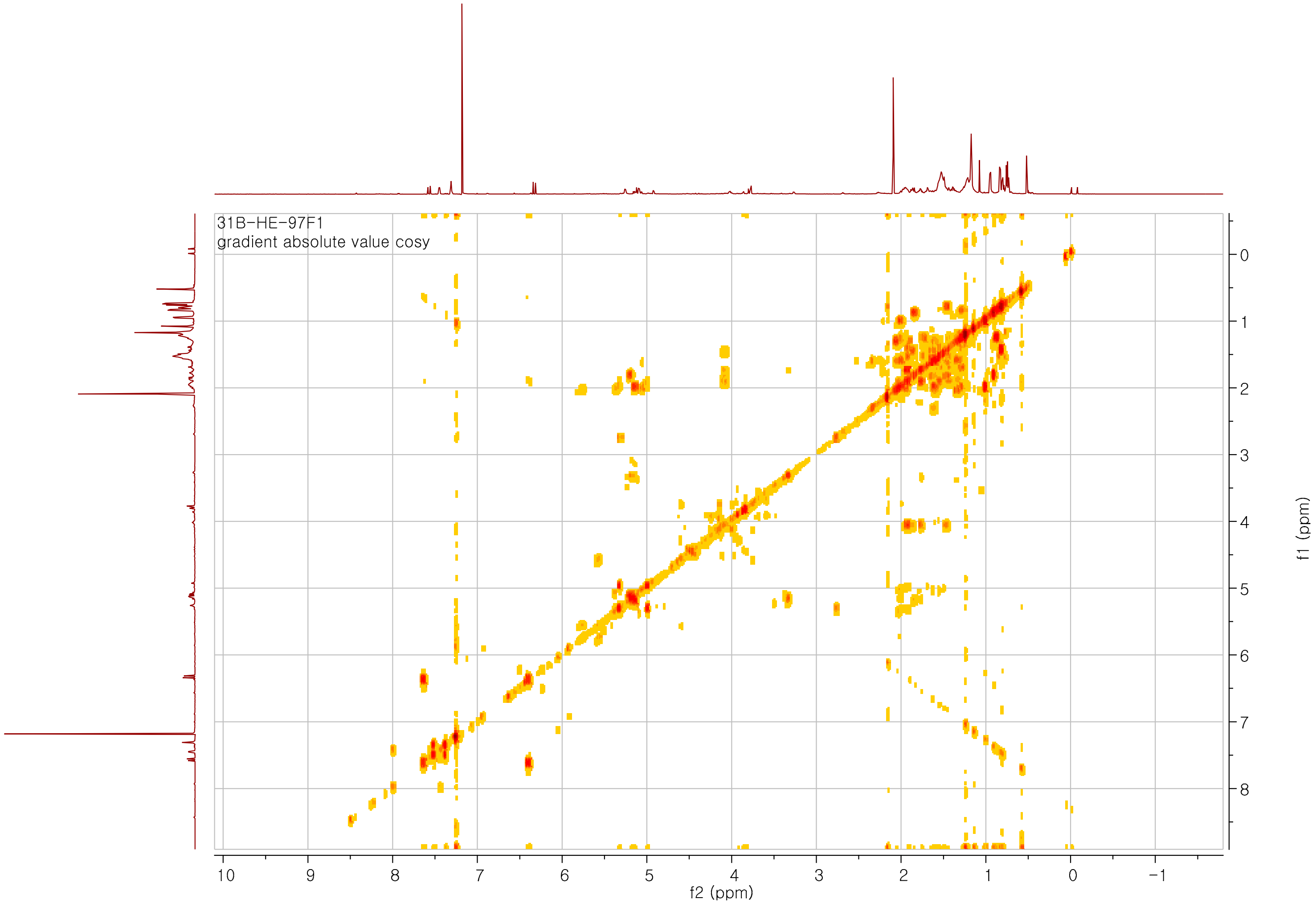

Figure S3. COSY spectrum of compound **6** ($CDCl_3$).

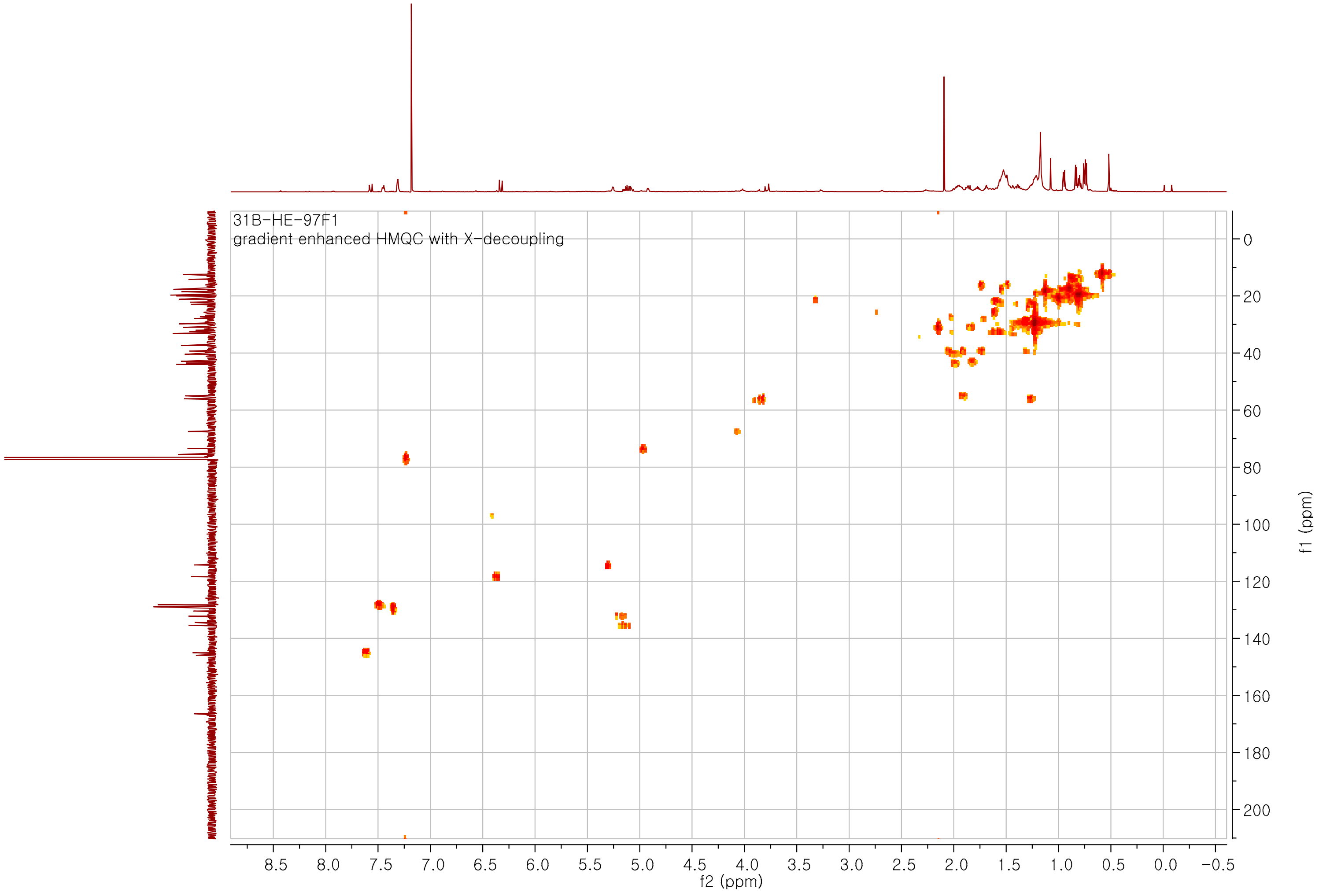

Figure S4. HMQC spectrum of compound **6** ($CDCl_3$).

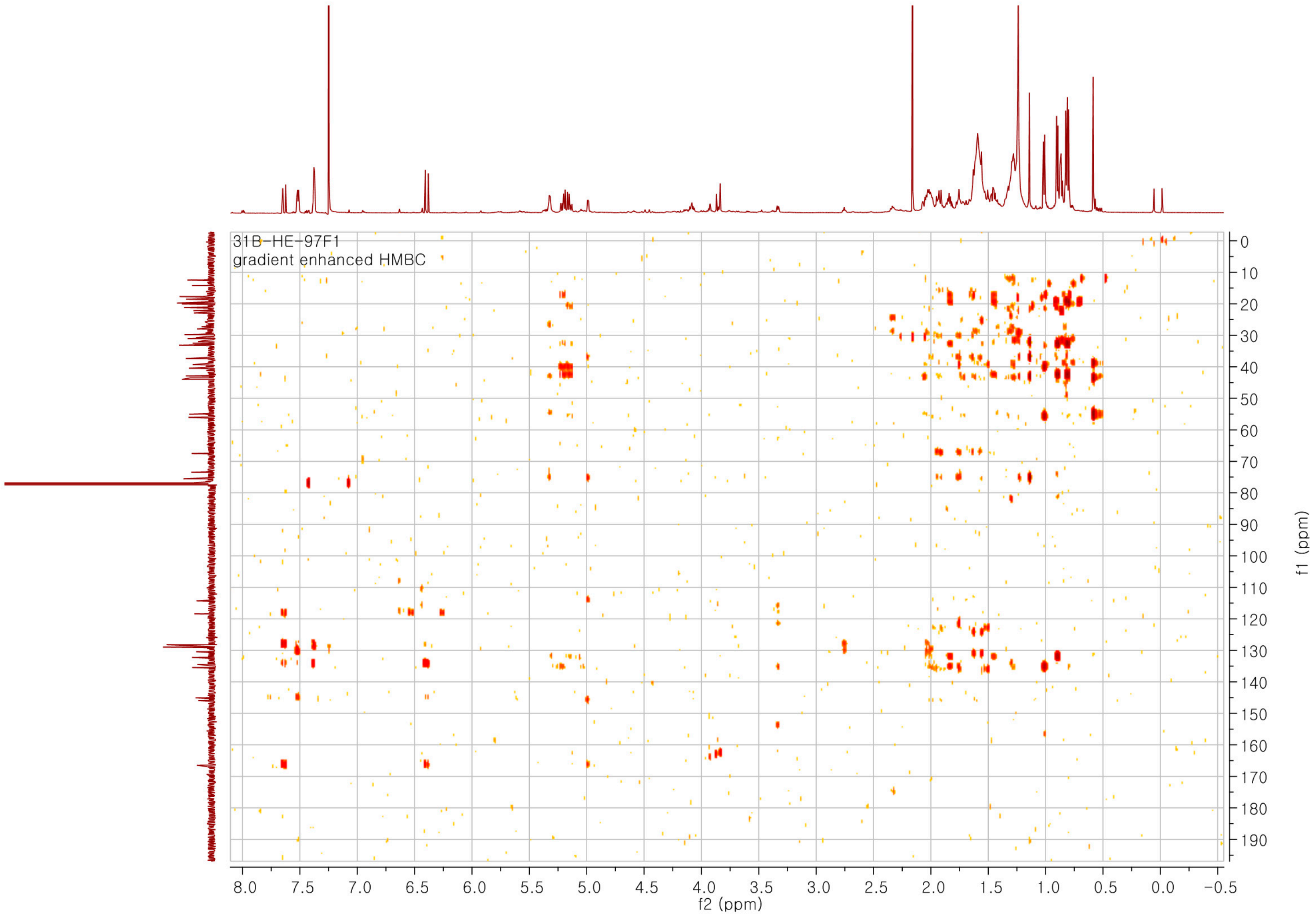

Figure S5. HMBC spectrum of compound **6** ($CDCl_3$).

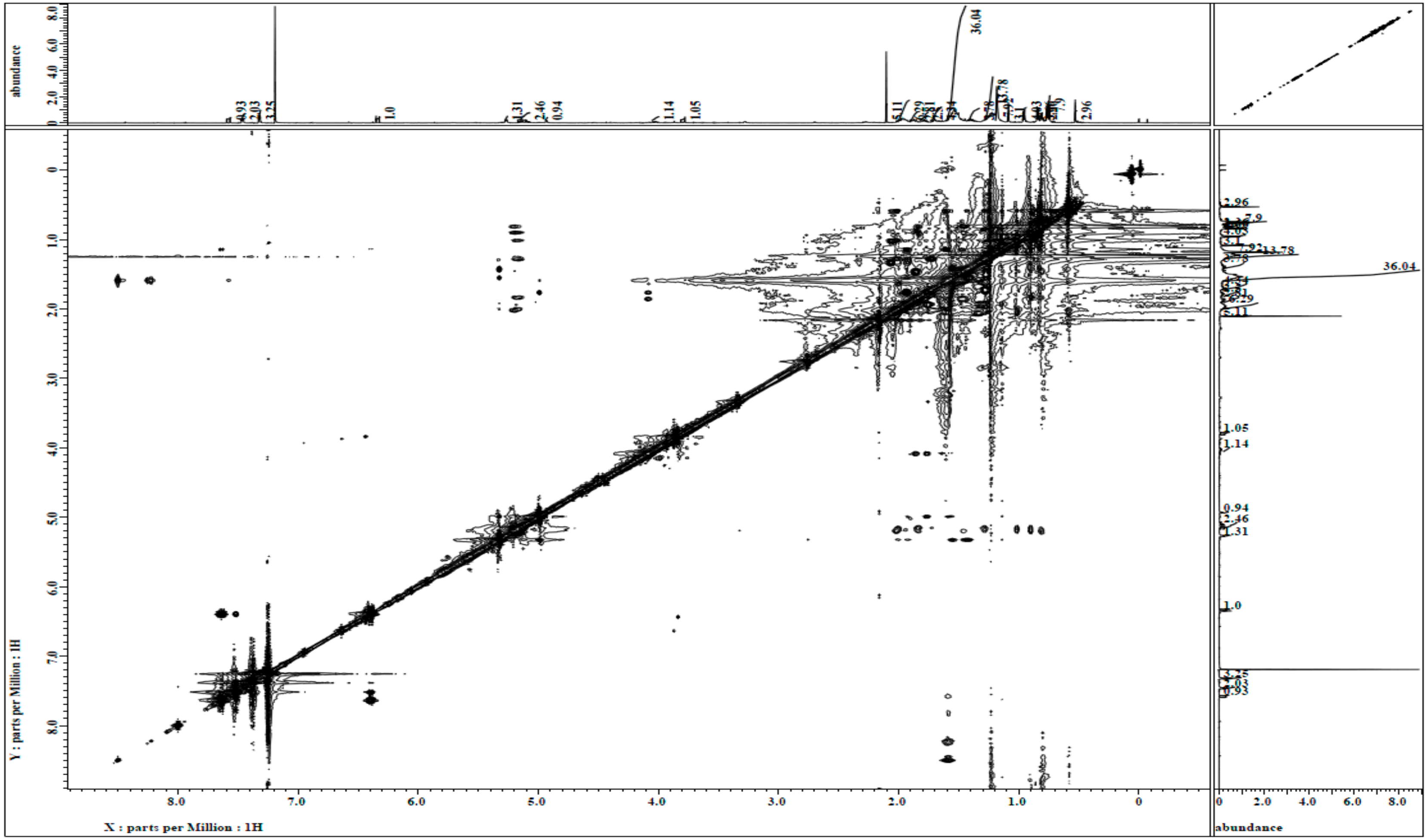

Figure S6. NOESY spectrum of compound **6** ($CDCl_3$).
